# The Effect of a Rotating Cone on Horseradish Peroxidase Aggregation on Mica Revealed by Atomic Force Microscopy

**DOI:** 10.3390/mi13111947

**Published:** 2022-11-10

**Authors:** Yuri D. Ivanov, Vadim Y. Tatur, Ivan D. Shumov, Andrey F. Kozlov, Anastasia A. Valueva, Irina A. Ivanova, Maria O. Ershova, Nina D. Ivanova, Igor N. Stepanov, Andrei A. Lukyanitsa, Vadim S. Ziborov

**Affiliations:** 1 Institute of Biomedical Chemistry, Pogodinskaya Str., 10 Build. 8, 119121 Moscow, Russia; 2 Joint Institute for High Temperatures of the Russian Academy of Sciences, 125412 Moscow, Russia; 3 Foundation of Perspective Technologies and Novations, 115682 Moscow, Russia; 4 Moscow State Academy of Veterinary Medicine and Biotechnology Named after Skryabin, 109472 Moscow, Russia; 5 Faculty of Computational Mathematics and Cybernetics, Moscow State University, 119991 Moscow, Russia

**Keywords:** horseradish peroxidase, enzyme aggregation, atomic force microscopy, triboelectric effect, enzyme-based biosensor

## Abstract

Our study reported herein aims to determine whether an electromagnetic field, induced triboelectrically by a metallic cone, rotating at a frequency of 167 Hz, has an effect on the properties of the horseradish peroxidase (HRP) enzyme. Atomic force microscopy (AFM) was employed to detect even the most subtle effects on single enzyme molecules. In parallel, a macroscopic method (spectrophotometry) was used to reveal whether the enzymatic activity of HRP in solution was affected. An aqueous solution of the enzyme was incubated at a distance of 2 cm from the rotating cone. The experiments were performed at various incubation times. The control experiments were performed with a non-rotating cone. The incubation of the HRP solution was found to cause the disaggregation of the enzyme. At longer incubation times, this disaggregation was found to be accompanied by the formation of higher-order aggregates; however, no change in the HRP enzymatic activity was observed. The results of our experiments could be of interest in the development of enzyme-based biosensors with rotating elements such as stirrers. Additionally, the results obtained herein are important for the correct interpretation of data obtained with such biosensors.

## 1. Introduction

Studies on the electrokinetic phenomena associated with triboelectric effects are currently receiving a good deal of attention. This effect involves the generation of an electric charge during the frictional contact between a liquid [1,2,3,4] (or a gas [5,6]) and a solid surface upon their motion. Importantly, the motion of a gas can also lead to the triboelectric effect [5] where the electric charge that is generated induces electromagnetic fields. This effect is essential in highly sensitive biosensor systems, in which a charge generated in the analyzed solution can significantly influence its use as a measurement tool. Nanowire biosensors are extremely sensitive to an electric charge and are often utilized in typical biosensor systems [7]. It has been reported that various elements with a conical shape are employed in biosensor systems [8,9], and a number of separators have conical elements or injectors with a liquid outlet in which swirling eddy currents can form. Furthermore, rotating elements are often used for stirring the analyzed solution in a biosensor [8,10]. With regard to biosensors, the triboelectric effect must be taken into account, since it can have significant effects on the properties of the biological macromolecules that are studied with these devices [11].

Enzymes represent a type of biological macromolecules that are often used in biosensor experiments [12]. The metabolic processes in living cells are regulated by enzymes [13]. Of these, peroxidases are well represented in plant and animal tissues [13] and they play important functional roles. They catalyze the oxidation of a broad spectrum of organic and inorganic compounds by hydrogen peroxide [14]. In the human body, the role of myeloperoxidase, which is involved in atherogenesis, should be noted [15]. Horseradish peroxidase (HRP) is widely employed as a model to study peroxidases [16,17]. Moreover, it is often used in enzyme-based biosensors [18,19]. Furthermore, the use of HRP for the detection of inorganic (heavy metal) ions [20,21] and organic compounds (phenols) [22] has been reported. This protein has already been characterized in considerable detail [23,24,25,26,27,28,29], which simplifies the explanation of any experimental results obtained by using it as a model. 

Many enzymes, including horseradish peroxidase, are known to form aggregates [23]. Changes in the enzyme aggregation state following external physical and chemical influences (e.g., electromagnetic, thermal, chemical influence, etc.) relate to changes in its spatial structure, which can lead to a pathological state. If this structural change does not affect the active site or chromophore groups of the enzyme, it is difficult to detect such a change by altering the kinetic parameters of the catalytic reaction. Accordingly, more sensitive methods are required for the detection of such changes in the enzyme structure. Atomic force microscopy (AFM) enables visualization at the level of single enzyme molecules [30,31,32], as was demonstrated in a study of the effect of knotted electromagnetic fields on enzyme properties [33]. Owing to its excellent height resolution [34], AFM reveals even the most subtle changes in the surface structure of visualized biological objects [35], including enzymes [36]. 

Herein, atomic force microscopy was employed to study the effect of a cone, rotating in air, on the aggregation state of an enzyme on mica using horseradish peroxidase as a model. To monitor the HRP adsorption properties and aggregation state after its exposure to the rotating cone, the height distribution of the adsorbed enzyme particles was plotted based on the AFM data. In addition, the HRP activity in a solution was estimated by the traditional spectrophotometric method. In our experiments, the aggregation state of HRP changed after the incubation of its solution near the apex of a rotating cone. Namely, the exposure to the rotating cone induced a shift in the height distribution maximum towards lower values, thus indicating the disaggregation of HRP. At longer incubation time, this disaggregation was accompanied by the formation of higher-order aggregates. In contrast, no effect from the stationary (non-rotating) cone was observed.

## 2. Materials and Methods

### 2.1. Chemicals and Enzyme

Peroxidase from horseradish, and its substrate 2,2′-azino-bis(3-ethylbenzothiazoline-6-sulfonate) (ABTS) were purchased from Sigma (St. Louis, MO, USA). Disodium hydrogen orthophosphate (Na_2_HPO_4_), citric acid and hydrogen peroxide (H_2_O_2_) were purchased from Reakhim (Moscow, Russia). All solutions were prepared using deionized ultrapure water (with 18.2 MOhms cm resistivity) obtained with a Simplicity UV system (Millipore, Molsheim, France).

The 0.1 µM enzyme solution used in the experiments was prepared by sequential ten-fold dilution of the initial 10 µM stock solution with 2 mM of Dulbecco’s modified phosphate buffered saline (PBSD; pH 7.4). The stock solution was prepared by dissolving 0.3 mg of the lyophilized enzyme powder in 0.7 mL of PBSD.

### 2.2. Experimental Setup

**Figure 1:**
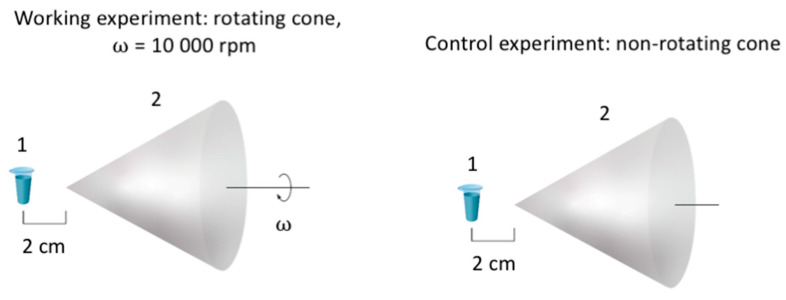
Experimental setup. The working experiments were performed with a cone rotating at 10,000 rpm (**left**), while the control experiments were performed with a non-rotating cone (**right**). The main elements of the setup are indicated by numbers: (1) the test tube with 1 mL of 0.1 µM of HRP solution in 2 mM PBSD (pH 7.4); (2) the rotating cone. The distance between the apex of the cone and the test tube was 2 cm.

In order to study the effect of a cone, rotating in air, on the HRP enzyme, the setup shown in Figure 1 was used. A test tube with 1 mL of enzyme solution (0.1 µM HRP in 2 mM of Dulbecco’s modified phosphate buffered saline, pH 7.4) was placed at a distance of 2 cm from the apex of a stainless-steel cone using a plastic holder, which allowed us to precisely define the 2 cm distance from the cone’s apex. In the working experiments, the cone rotated clockwise at 10,000 rpm. In the control experiments, the enzyme sample was incubated near the non-rotating cone at the same distance (2 cm). In the experiments, the enzyme sample was incubated near the apex of the cone for either 10 or 20 min at a room temperature of 17 °C. The test tube was placed on an anti-vibration table, thus excluding the influence of vibrations from the rotating cone on the enzyme.

The cone’s base diameter was 115 mm, and its apex angle was 51°. The cone material was stainless steel.

After the incubation, the enzyme solution was subjected to analysis by AFM (to determine the adsorption properties and aggregation state of the enzyme on mica) and by spectrophotometry (to estimate the enzymatic activity).

### 2.3. Atomic Force Microscopy

The AFM samples were prepared using the direct surface adsorption method reported by Kiselyova et al. [37]. Freshly cleaved muscovite mica sheets (SPI, West Chester, PA, USA) were used as AFM substrates. To prepare the samples, a 7 × 15 mm mica sheet was immersed in 800 μL of either of the 0.1 µM HRP sample solutions, and incubated in a shaker at 600 rpm for 10 min at a constant temperature of 25 °C. Then, each mica sheet was rinsed with ultrapure water and dried in an airflow. 

The use of the 0.1 µM HRP solution for the direct surface adsorption experiments with bare mica allowed us to image isolated enzyme particles on the substrate surface. In contrast, at higher (micromolar) enzyme concentrations, it forms continuous layers on the substrate surface, which makes it impossible to obtain an accurate counting of the adsorbed objects, and the aggregation state. This is consistent with the results reported by Ignatenko et al. [23]. Using dynamic light scattering, these authors revealed that native HRP is prone to aggregation in micromolar solutions, forming large (up to 150 nm) aggregates [23]. At low (10 nM and 1 nM) concentrations, we did not observe HRP adsorption on the bare mica [33].

The so-prepared mica substrate with adsorbed HRP particles was then scanned with a Titanium multimode atomic force microscope (NT-MDT, Zelenograd, Russia; the microscope belongs to the equipment of “Human Proteome” Core Facility of the Institute of Biomedical Chemistry, supported by the Ministry of Education and Science of Russian Federation, agreement 14.621.21.0017, unique project ID: RFMEFI62117x0017) in tapping mode in air with the use of NSG03 cantilevers (“TipsNano”, Zelenograd, Russia). The resonant frequency of the cantilevers was 47–150 kHz, and the force constant was 0.35–6.1 N/m. The calibration of the microscope by height was done on a TGZ1 calibration grating (NT-MDT, Zelenograd, Russia) with a step height of 21.4 ± 1.5 nm. The total amount of measured particles in each sample was >200, and the number of frames for each sample was ≥10. In each AFM experiment, no less than three technical replicates were performed for each enzyme sample studied. The relative distributions of the visualized particles based on height *ρ*(*h*) were calculated using the software developed at the Institute of Biomedical Chemistry in collaboration with the Foundation of Perspective Technologies and Novations as described by Pleshakova et al. [38]. In blank experiments performed with enzyme-free buffer, no objects with heights > 0.5 nm were detected.

### 2.4. Spectrophotometry

HRP activity was estimated according to the technique described in detail by Sanders et al. using ABTS as the substrate in phosphate-citrate buffer [39] at pH 5.0 [39,40], as described in our previous papers [11,33,36,41,42], with an Agilent 8453 UV-visible spectrophotometer (Agilent Technologies Deutschland GmbH, Waldbronn, Germany). Spectrophotometry measurements for each sample were repeated at least three times.

## 3. Results

### 3.1. Atomic Force Microscopy

**Figure 2:**
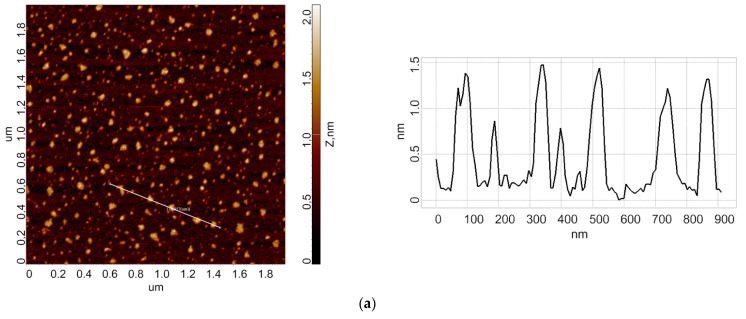

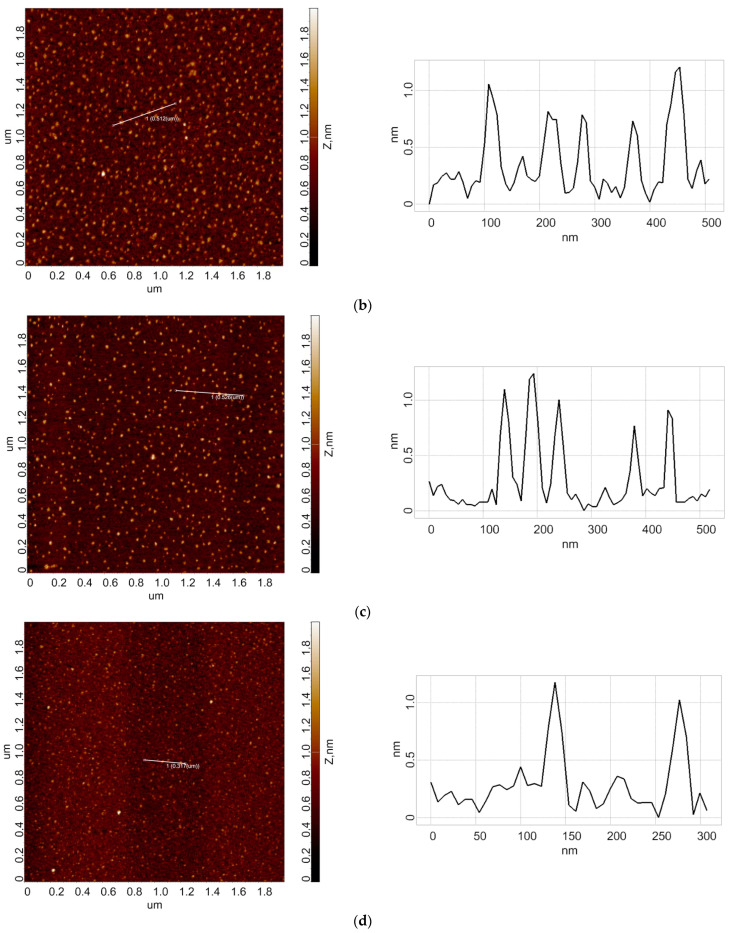
Typical AFM images of mica surface with adsorbed HRP (left) and their respective cross section profiles (right) obtained for working and control HRP solutions. Experimental conditions: *w* = 0 (non-rotating cone), *t* = 10 min (control) (**a**); *w* = 10,000 (rotating cone), *t* = 10 min (working experiment) (**b**); *w* = 0 (non-rotating cone), *t* = 20 min (control) (**c**); *w* = 10,000 rpm (rotating cone), *t* = 20 min (working experiment) (**d**). For all AFM images presented, the scan size was 2 µm × 2 µm, and the Z scale was from 0 to 2 nm.

In the first step, control experiments were performed in order to obtain the height distribution of HRP biomolecules after the samples were incubated near the non-rotating cone. In the second step, working experiments were performed in order to obtain the height distribution of HRP biomolecules after the samples were incubated near the cone, which was rotating clockwise at 10,000 rpm. Figure 2 displays typical AFM images obtained from the experiments.

**Figure 3:**
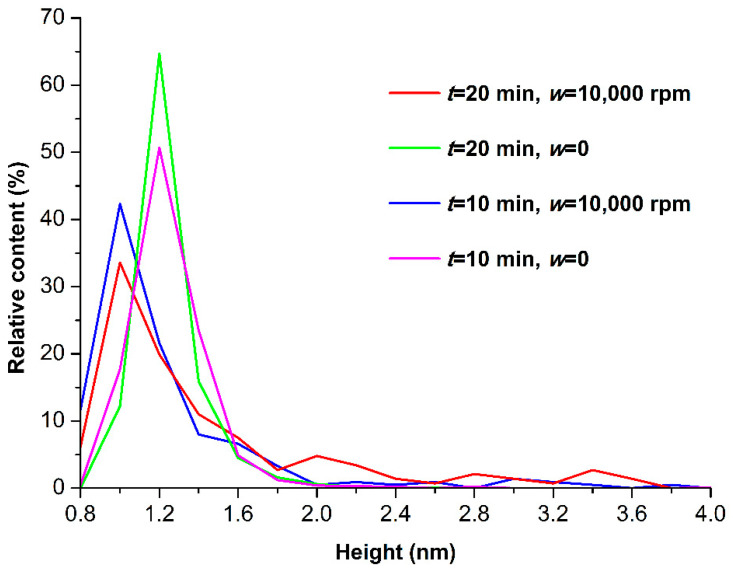
Relative distributions of the mica-adsorbed HRP particles with height *ρ*(*h*) obtained for the HRP samples incubated at 2 cm from the cone. Experimental conditions: *w* = 0 (non-rotating cone), *t* = 10 min (control) (magenta); *w* = 10,000 rpm (rotating cone), *t* = 10 min (working experiment) (blue); *w* = 0 (non-rotating cone), *t* = 20 min (control) (green); *w* = 10,000 rpm (rotating cone), *t* = 20 min (working experiment) (red).

As can be seen from Figure 2a,c, in the case of a control HRP sample, the enzyme adsorbs on the mica surface in the form of compact objects. A cross section of typical AFM-visualized objects with a height of approximately 1.2 nm is also presented. The AFM data obtained in the control experiments were processed, and then the *ρ*(*h*) distribution was plotted. Figure 3 displays the height distributions obtained after processing the AFM data.

Analysis of the *ρ*(*h*) distributions obtained for the control HRP sample indicates that in the control experiments with the non-rotating cone (*w* = 0) at 10 min incubation time, the maximum of the distribution of the AFM-visualized objects with regard to height corresponds to *h_max_* = 1.2 nm. In the working experiments with the cone rotating at *w* = 10,000 rpm, the *ρ*(*h*)*_max_* shifts to the left by 0.2 nm. This clearly indicates the disaggregation of mica-adsorbed HRP. At the same time, in these working experiments, there is an increase in the content of objects with *h* ≈ 1.6 nm contributing to the right wing of the *ρ*(*h*) distribution—as compared with the control experiments performed at 10 min incubation time.

Furthermore, at longer incubation time (*t* = 20 min), the *h_max_* remains unchanged in the control experiments, while in the working experiments the *h_max_* shifts to 1.0 nm, clearly indicating HRP disaggregation. At the same time, in the working experiments at *t* = 20 min, the content of mica-adsorbed particles with *h* > 1.8 nm, contributing to the right wing of the *ρ*(*h*) distribution, is considerably more significant in comparison with that observed at shorter incubation times. An increase in the content of objects with *h* > 1.6 nm indicates an increase in the HRP aggregation on mica. Thus, in our experiments, HRP disaggregation occurs in parallel with the formation of higher-order aggregates, which were not observed in the control experiments with the non-rotating cone.

### 3.2. Spectrophotometry

**Figure 4:**
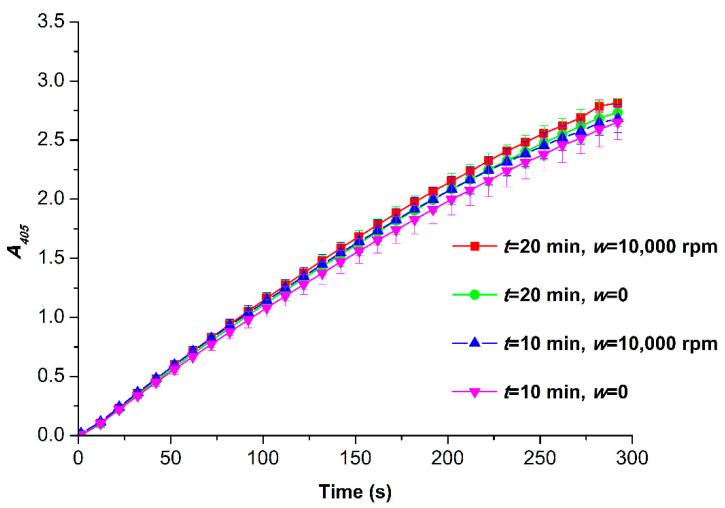
*A*_405_(*t*) kinetic curves obtained using the HRP-ABTS-H_2_O_2_ system for HRP samples incubated at *w* = 0 (non-rotating cone), *t* = 10 min (control) (magenta); *w* = 10,000 rpm (rotating cone), *t* = 10 min (working experiment) (blue); *w* = 0 (non-rotating cone), *t* = 20 min (control) (green); *w* = 10,000 rpm (rotating cone), *t* = 20 min (working experiment) (red). Measurement conditions: HRP:ABTS:H_2_O_2_ = 1 nM:2.5 mM:0.3 mM; pH 5.0; cell pathlength 1 cm, temperature 25 °C.

The HRP activity was estimated for all the samples studied by AFM. Figure 4 displays typical *A*_405_(*t*) kinetic curves obtained in both the control and the working experiments.

The data presented in Figure 4 were compared based on the least square method as described in our recent paper [42]. The curves in Figure 4 indicate that there is no difference in the enzymatic activity of HRP in the samples studied. In our experiments, the field frequency was 167 Hz. This value is comparable to the 100 Hz frequency, for which no effect on the enzymatic activity of HRP was reported by Caliga et al. [43]. Accordingly, the data from the experiments performed by us and by Caliga et al. are in a good agreement with respect to the relationship between the unaffected enzymatic activity and the low-frequency electromagnetic field (LFEF) frequency. This is how we explain the absence of the effect of LFEF on the enzymatic activity in our experiments.

## 4. Discussion

Herein, the effects of a cone, rotating in air, on the properties of HRP enzyme were investigated. The incubation of the enzyme solution near the rotating cone was found to cause a change in the distribution of mica-adsorbed HRP with heights that were comparative to those obtained for the control enzyme samples incubated near the non-rotating cone.

In the control experiments with the non-rotating cone, the maximum number of HRP particles adsorbed from the control HRP samples on to mica, had a height *h_max_* = 1.2 nm, while their width at half-height was 0.2 nm. In contrast, in the working experiments with the rotating cone, the majority of mica-adsorbed HRP particles had a height *h_max_* = 1.0 nm. That is, the maximum of the *ρ*(*h*) distribution obtained in the working experiments, shifted left by 0.2 nm in comparison with that obtained in the control experiments, indicating the disaggregation of the enzyme. In the working experiments performed for a longer incubation time (*t* = 20 min), a considerable increase in the content of mica-adsorbed particles with *h* > 1.8 nm was observed, indicating the formation of higher-order enzyme aggregates. Based on these findings, one can conclude that in our experiments, exposure of the HRP sample to the rotating cone induced a disaggregation of the enzyme upon its adsorption on mica and at longer exposures, this disaggregation occurs in parallel with the formation of higher-order aggregates. The enzymatic activity of HRP remained unchanged.

Such an effect of the cone rotation on the HRP adsorption properties can be explained as follows. Water represents a non-equilibrium mixture of ortho- and para-H_2_O isomers [44]. The ratio between these isomers changes in the presence of the HRP enzyme (in comparison with the enzyme-free aqueous solution). This can be explained in the following way. Pershin [45] reported that the data on H_2_O molecules indicates two basic differences that manifest themselves in anomalous properties of water. One of these properties provides the stable formation of ordered ice-like structures whereas another factor favors the formation of disordered complexes. Rearrangement of these molecules in the latter complexes, and a change in the number of hydrogen bonds between them occurs within the time interval of proton transition, and they exist in equilibrium with the ordered structures. The only distinctive feature of H_2_O is the quantum differences between the spin isomers of H_2_O. Pershin [45] justified the quantum nature of the anomalous properties of water, which was determined by the existence of ortho- and para-H_2_O isomers. They differ by their quantum parameters (spin state, magnetic moment, and rotational spectra). In [46,47], the rotational spectra of water obtained by four-photon coherent spectroscopy allowed us to build a new concept of water as a mixture of molecular complexes. The formation of mixtures of molecular complexes is more preferable for para-H_2_O than for ortho-H_2_O, which is always rotating. Accordingly, para-H_2_O is formed with higher probability. Furthermore, in [48], the splitting of the OH-band in the Raman spectra into two components was found to be separated by a narrow (~200 cm^−1^ [48]) band gap. These observed fluctuations in the Raman spectra occur at the expense of ortho-/para-conversion of the H_2_O isomers, whose spectral lines were detected in [46]. It is notable that at room temperature (20–25 °C), there was a coincidence in the energy for the ortho- and para-H_2_O isomers. Based on this, Artmann et al. [49] demonstrated that the structure of the hydration shell of hemoglobin is predominantly formed by para-H_2_O. Moreover, the presence of an enzyme in solution leads to a shift in the ratio between ortho- and para-H_2_O isomers, as was demonstrated with the example of chimotrypsin by Bunkin et al. [47]. Enzyme hydration shells consist of the adsorbed para-isomers of water [50]. Accordingly, such a shift is supposed to take place in the case of other enzymes, and this is how we explain the phenomenon observed in our experiments.

Furthermore, exposure to an electromagnetic field of low intensity can lead to ortho/para transitions [51]. Even low-intensity electric and electromagnetic fields substantially alter the ratio between ortho- and para-H_2_O isomers [47,51,52]. This concept is applicable to low-energy impacts (whose energy *E* << *kT*), and microwave fields, and LFEFs fulfil this requirement. Lednev [53,54] justified the impact of extremely weak LFEF (whose frequency ranges from several Hz to several kHz) on biological objects. Moreover, in several papers, the action of LFEF on enzymes was demonstrated to have a varied effect, either stimulating, not influencing, or suppressing their enzymatic activity. For a number of membrane-associated enzymes (alkaline phosphatase, acetylcholinesterase from blood cell membranes, acetylcholinesterase from synaptosomes, phosphoglycerate kinase and adenylate kinase), Morelli et al. reported a decrease in their activity under the action of a 75 Hz LFEF, while other enzymes (CaATPase, Na/K ATPase, and succinic dehydrogenase) were found to be virtually insensitive to such an action [55]. A two-fold increase in the activity of cAMP-dependent protein kinase in human skin fibroblasts was observed by Thumm et al. after 1 h exposure to 20 Hz LFEF (7–8 mT) [56]. Caliga et al. [43] observed a nearly two-fold decrease in the catalytic efficiency of HRP after its exposure to a 50 Hz (2.7 mT) LFEF, while a 100 Hz (5.5 mT) LFEF had virtually no effect on the enzyme—this is another important example of how the effect of LFEF on an enzyme can vary depending on the field parameters. In our experiments, the field frequency was 167 Hz. This value is comparable to the 100 Hz frequency, for which no effect on the enzymatic activity of HRP was reported by Caliga et al. Nevertheless, according to Pershin, the frequency of just several Hz is sufficient to affect the ratio between ortho- and para-H_2_O isomers [51]. Although the enzymatic activity remained unaffected in our experiments, the enzyme hydration shells were nevertheless affected; thus, there was a change in the enzyme aggregation state on mica. The latter is determined by the interaction between the enzyme molecules, and by the interactions of the enzyme molecules with the substrate surface and the solvent [41]. The most comprehensive and correct way is to consider all these interactions in connection with each other. In other words, each type of interaction could well influence the other types of interactions. Since there was no change in the enzymatic activity in solution, only the enzyme aggregation/disaggregation on the substrate surface was affected. Therefore, we conclude that the enzyme–enzyme interactions should be considered together with the enzyme–substrate interactions. As regards the latter, it should be noted that in general, the surface of a biomolecule bears binding sites of all the following types: hydrophilic (but uncharged), hydrophobic, and electrostatically charged [57,58]. Despite this fact, one particular type of interaction of a biomolecule with a substrate could well be preferable. A good example of this is antifreeze glycoprotein fraction 8, which prefers adsorption in the hydrophilic regions of its substrate surface even though its globule bears multiple binding sites [58].

As regards enzyme–solvent interactions, this is what is called “hydration” in the case of aqueous solutions, and this was discussed above. Hydration shells can well influence enzymes’ structure [59,60] and activity [60] (the latter is not the case in our experiments). In general, organic solvents can also act instead of water, replacing it at the functional sites of an enzyme and loosening its structure, thus performing the solvation function [61], as was demonstrated for cytochrome *c* [62].

A change in the electromagnetic field in the vicinity of the cone, which rotates with a frequency of 167 Hz, can occur at the expense of friction occurring in air. Namely, air flow near the cone surface leads to the electrification of air due to the well-described triboelectric effect mentioned in the Introduction. The so-generated charged particles induce an electromagnetic field. This field, in its turn, can induce a change in the ratio between ortho- and para-H_2_O isomers in an aqueous enzyme solution, thus altering its hydration. Thus, this alteration in the enzyme hydration leads to a change in the enzyme aggregation on mica as discussed above.

The results of our experiments should be considered when using enzymes in bioreactors and biosensors with rotating elements such as stirrers. Of course, other external factors should also be taken into account—for instance, temperature represents another factor influencing HRP aggregation [63]. Accordingly, the results obtained herein can be helpful for the correct interpretation of data obtained with such biosensors. Our results are also useful for modeling the pathological processes associated with enzymes involved in the formation of functionally significant multiprotein complexes (for instance, inflammatory processes involving myeloperoxidase, which exists in these complexes in the form of dimers [64]). In addition, the complex process of protein disaggregation and aggregation can cause changes in hemodynamics in small vessels, but at the same time, it can affect pathological changes associated with the functioning of enzymes in the body [65].

## 5. Conclusions

The influence of a cone, rotating in air with a high angular velocity, on the HRP enzyme was studied using AFM. The incubation of aqueous HRP solution in the vicinity of the rotating cone was found to affect the adsorption properties of the enzyme on mica, while the enzymatic activity of HRP remained unaffected. The results of our experiments should be considered when using enzymes in bioreactors and biosensors with rotating elements such as stirrers. Accordingly, the results obtained herein are important for the correct interpretation of data obtained with such biosensors.

## Data Availability

Correspondence and requests for materials should be addressed to Y.D.I.
